# Comparison of twelve liver functional reserve models for outcome prediction in patients with hepatocellular carcinoma undergoing surgical resection

**DOI:** 10.1038/s41598-018-22923-4

**Published:** 2018-03-19

**Authors:** Shu-Yein Ho, Po-Hong Liu, Chia-Yang Hsu, Cheng-Yuan Hsia, Chien-Wei Su, Yun-Hsuan Lee, Yi-Hsiang Huang, Fa-Yauh Lee, Ming-Chih Hou, Teh-Ia Huo

**Affiliations:** 1 Department of Medicine, Taipei Veterans General Hospital, Taipei, Taiwan; 2 Department of Surgery, Taipei Veterans General Hospital, Taipei, Taiwan; 3 Faculty of Medicine, National Yang-Ming University School of Medicine, Taipei, Taiwan; 4 Institute of Clinical Medicine, National Yang-Ming University School of Medicine, Taipei, Taiwan; 5 Institute of Pharmacology, National Yang-Ming University School of Medicine, Taipei, Taiwan; 6 Harvard T.H. Chan School of Public Health, Boston, MA USA; 7 Department of Internal Medicine, University of Nevada School of Medicine, Reno, NV USA

## Abstract

Various noninvasive liver functional reserve models have been proposed, but their prognostic ability in patients with hepatocellular carcinoma (HCC) is unclear. We aimed to investigate the performance of twelve noninvasive liver reserve models in HCC patients undergoing surgical resection. A total of 645 patients undergoing resection were prospectively identified and retrospectively analyzed. Tumor recurrence, overall survival, and independent prognostic factors were evaluated by the Cox proportional hazards model. Of the twelve models, the King’s score showed the highest homogeneity and lowest corrected Akaike information criterion (AICc) value, suggesting a better predictive ability for tumor recurrence. In multivariate Cox analysis, we confirmed that King’s score, tumor size and serum alpha-fetoprotein level were independent predictors associated with recurrence. In survival prediction, albumin-bilirubin (ALBI) revealed the highest homogeneity and lowest value among twelve invasive models, indicating a better prognostic performance. In the Cox model, ALBI grade, tumor burden, alpha-fetoprotein, vascular invasion, diabetes mellitus and performance status were independent predictors linked with overall survival. In summary, the currently used liver function models have differential predictive ability for HCC patients undergoing surgical resection. The King’s score is a feasible tool to predict tumor recurrence, whereas ALBI grade is a more robust model for prognostic prediction.

## Introduction

Hepatocellular carcinoma (HCC) is one of the most common malignancies worldwide, accounting for more the 700,000 deaths each year^1^. Chronic hepatitis B and C virus (HBV, HCV) infection and alcoholism are the major risk factors for HCC^2^. HCC develops in the background of chronic liver disease or cirrhosis in 70–90% patients, and various degrees of liver dysfunction are usually present at the time of diagnosis. Surgical resection is the curative treatment option for patients with early stage HCC with well preserved liver function, and provides 5-year survival rate up to 70%^3,4^. However, tumor recurrence after surgery is common and is associated with a decreased overall survival^5,6^.

The prognosis and management of HCC is typically influenced by tumor burden, liver functional reserve and performance status^3^. The Child-Turcotte-Pugh (CTP) classification has been widely used for decades in assessing the severity of liver dysfunction. Many HCC staging systems, including the Barcelona Clínic Liver Cancer (BCLC) staging system, utilize CTP classification as an indicator of liver disease severity. However, the CTP classification is not an evidence-based practice. The model for end-stage liver disease (MELD) has been a prevailing system to prioritize cirrhotic patients awaiting liver transplantation^7^, and is used in assessing liver functional reserve and outcome for HCC patients^8–10^. Another important marker, indocyanine green retention rate at 15 minutes (ICG-15) test, has also been widely used to evaluate liver reserve in surgical HCC patients^11^.

Alternatively, the albumin-bilirubin (ALBI) and the platelet-albumin-bilirubin (PALBI) grade were recently proposed to assess liver functional reserve in HCC^12,13^. In addition, serum sodium concentration was found to inversely correlate with the severity of cirrhosis, and has been used to assess the degree of portal hypertension^14–16^. Other tools to evaluate liver functional reserve include aspartate aminotransferase-to-platelet ratio (APRI), fibrosis index based on 4 factors (FIB-4), King’s score, cirrhosis discriminate index (CDS), Lok index and the Göteborg University Cirrhosis Index (GUCI)^17–22^. These models incorporate different clinical parameters such as age and serum biochemistries. Up to date, twelve noninvasive liver reserve models are used to assess the degree of liver dysfunction, but the prognostic role of these models in HCC patients remains unclear. This study aimed to investigate the correlation of these noninvasive models and their prognostic impact on tumor recurrence and overall survival in HCC patients undergoing surgical resection.

## Patients and Methods

### Patients and follow-up

During a 12-year period between 2003 to 2015, patients with newly diagnosed HCC and admitted to Taipei Veterans General Hospital were prospectively identified and retrospectively analyzed. A total 645 patients undergoing surgical resection were enrolled in this study. The baseline demographics, etiology of liver disease, tumor status and serum biochemistries were collected at the time of diagnosis. Tumor recurrence, subsequent anti-cancer therapy, and overall survival were recorded. The inclusion criteria of surgery were (1) tumor involving no more than three Couinaud segments, (2) CTP class A or B and data for ICG-15, (3) no main portal vein trunk involvement or distant metastases, and (4) absence of other major diseases that may complicate resection^23^.

After surgery, the patients were followed up with imaging studies and serum a-fetoprotein (AFP) level every 3 to 6 months until death or dropout from the follow-up program. This study complies with the standard of the Declaration of Helsinki and current ethical guidelines, and has been approved by the Institutional Review Broad of Taipei Veterans General Hospital. Waiver of consent was obtained, and patient records/information was anonymized and de-identified prior to analysis.

### Diagnosis and definition

The pre-operative diagnosis of HCC was histologically confirmed or based on the findings of typical four-phase multidetector contrast-enhanced dynamic computed tomography (CT) scan or magnetic resonance imaging (MRI)^3^. The performance status was assessed by using the Eastern Cooperative Oncology Group Performance scaling ranging from 0 (asymptomatic) to 4 (confined to bed). Intrahepatic recurrence was defined as residual disease within or adjacent to the previously treated tumor site, whereas extrahepatic recurrence was defined as emergence of the tumor elsewhere in or outside the liver^24^.

### Treatment

Surgical resection was performed by our experienced surgical team. The operative procedures have been previously described in detail^25–27^. The resected liver tissue was sent for gross and microscopic examinations, and the recorded tumor size was based on the largest dimension of the resected specimen. The treatment of recurrence HCC included re-resection, local ablative treatment, transarterial chemoembolization, targeted therapy, chemotherapy, radiotherapy and best supportive care.

### Grading of 12 models

The calculation of 12 noninvasive liver functional reserve models was based on clinical variables and serum biochemistries at the time of diagnosis. The grading of these liver functional reserve models was according to published studies^8–10,12,13,17–21,28,29^. Grade 1 indicates adequate liver functions, and grade 3 is associated with poor liver reserve (Table 1).

**Table 1:** Formula and grading of 12 noninvasive liver functional reserve models.

Noninvasive blood testing for liver serve makers	Formula		
ALBI, Grade 1/2/3 (<−2.6/−2.6–≤−1.39 />−1.39)	(log(Bilirubin[μmol/L]) × 0.66) + (Albumin[g/L] × −0.085)		
APRI, Grade 1/2/3 (<0.5/0.5–1.5/>1.5)	[(AST/upper limit of normal)/Platelet Count (10^9^/l)] × 100		
CTP, A/B/C, grade 1/2/3/ (5–6/7–9/10–15)	Encephalopathy: none = 1, grade 1 or 2 = 2, grade 3 or 4 = 3 Ascites: none = 1, mild to moderate = 2, severe = 3 Bilirubin(mg/dl): <2 = 1, 2–3 = 2, >3 = 3 Albumin(g/dl): >3.5 = 1, 2.8–3.5 = 2, <2.8 = 3 PT sec (INR): <4 (1.7) = 1, 4–6 (1.7–2.3) = 2, >6 (>2.3) = 3		
CDS, Grade 1/2/3 (<4/4–7/>7)	Platelet count ( × 10^9^/L): >340 = 0; 280–339 = 1; 220–279 = 2; 160–219 = 3; 100–159 = 4; 40–99 = 5; <40 = 6	ALT/AST ratio: >1.7 = 0; 1.2–1.7 = 1; 0.6–1.19 = 2; <0.6 = 3	INR: <1.1 = 0; 1.1–1.4 = 1; >1.4 = 2 CDS is the sum of the above (possible value 0–11)
FIB-4 index, Grade 1/2/3 (<1.45/1.45–3.25/>3.25)	(Age[years] × AST[U/L])/(platelet [10^9^] × ALT[U/L]^1/2^)		
GUCI, Grade 1/2/3 (<0.5/0.5–1.56/>1.56)	[AST/TOPNORMAL AST] × INR × 100/(Platelets × 10^9^)		
Lok index, Grade 1/2/3 (<0.5/0.5–0.8/>0.8)	Lok Index = e^(LogOddsLok)^/(1 + e^(LogOddsLok)^) Log Odds Lok = (1.26 × AST/ALT) + (5.27 × INR) − (0.0089 × Platelets x10^9^) − 5.56		
MELD, Grade 1/2/3 (<8/8–12/>12)	10 × ((0.957 × ln(Creatinine)) + (0.378 × ln(Bilirubin)) + (1.12 × ln(INR))) + 6.43		
PABLI, Grade1/2/3 (≤−2.53, −2.53 and ≤−2.09, >−2.09)	(2.02 × log_10_ bilirubin) − [0.37 × (log_10_ bilirubin(umol/L))^2]^ − 0.04 × albumin (g/L) − 3.48 × log_10_ platelets(10^9^/L) + 1.01 × (log_10_ platelets(10^9^/l))^2^		
King’s score (<7.6/7.6–16.7/16.7)	Age × AST × INR/[platelets (10^9^/l)]		
Serum sodium (≦135/>135 mmole/L)			
ICG-15 test (%) (10/10–20/>20)			

### Statistical Analysis

All statistical analyses were conducted using the SPSS for Windows version 21 release (SPSS Inc., Chicago, IL, USA). The *X*^2^ test or Fisher’s exact test was used to analyze categorical variables and the Mann-Whitney ranked sum test for continuous variables. The recurrence-free survival and overall survival were estimated by the Kaplan-Meier method and compared by a log-rank test. Independent prognostic factors that were possibly linked to recurrence-free survival and overall survival were analyzed. Factors that were significant in the univariate analysis were entered into the adjusted multivariate Cox proportional hazards model to determine adjusted hazard ratio (HR) and 95% confidence interval (CI)^30^.

The discriminatory ability of different models associated with tumor recurrence and overall survival was examined by using the Cox proportional hazards model, and the consequences of the Cox model were expressed with the corrected Akaike information criterion (AICc), which reveals how the model affects the dependent variable and represents an overall assessment of the model^31,32^. The lower the AIC, the more explanatory and informative the model is^33^. We also examined the correlation of ICG-15 and other 11 noninvasive liver functional reserve models. For all tests, a p < 0.05 was considered statistically significant.

## Results

### Baseline characteristics

A prospective data set of 645 patients who received surgical resection as curative treatment were enrolled during the study period. Baseline demographics and clinical information of these patients are shown in Table 2. The median age was 61 years with the majority being male (80%). Three hundred and twenty-seven (51%) patients had HBV infection, and 131(20%) had a history of diabetes mellitus. Two hundred and thirty (36%) patients had tumor size ≤3 cm and 633 (98%) of had ≤3 nodules at initial presentation. In these patients, 254 (39%) and 232 (36%) received lobectomy and bi-segmentectomy respectively, while 133 (21%) and 26 (4%) patients received segmentectomy and sub-segmentectomy respectively. All patients were histologically confirmed HCC and were free of surgical margin.

**Table 2:** Baseline characteristics of hepatocellular carcinoma undergoing surgical resection.

Variables	Patients (n = 645)
Age (years, median [IQR])	61[52–70]
Male, n (%)	518 (80)
Etiologies of liver disease	
HBV, n (%)	327 (51)
HCV, n (%)	116(18)
HBV + HCV, n (%)	27 (4)
Alcohol, n (%)	19 (3)
Diabetes mellitus, n (%)	131 (20)
Performance status (0/1/2–4), n (%)	507/104/34 (79/15/6)
Ascites, n (%)	45 (7)
ICG (%, median [IQR])	10 [6–14]
Apha-fetoprotein (ng/mL) median [IQR]	25.7 [7.27–301]
Tumor nodules (≦3/>3), n (%)	633/12 (98/2)
Maximal tumor diameter (≤3/>3 cm), n(%)	230/415(36/64)
Vascular invasion, n (%)	68 (10%)
ALT (IU/L), median [IQR]	45 [29–75.5]
AST (IU/L), median [IQR]	44 [29–73.50]
Laboratory values (mean ± SD)	
Alkaline phosphatase (IU/L)	99.80 ± 78.49
Albumin (g/L)	39.93 ± 5.03
Total bilirubin (μmol/L)	14.41 ± 10.38
Creatinine (mg/dl)	1.05 ± 0.74
Platelets (1,000/μL)	180.96 ± 82.00
INR of prothrombin time (sec)	1.03 ± 0.11
Serum sodium (mmol/L)	139.59 ± 2.75
Non-invasive liver functional reserve models	
ALBI grade (1/2/3), n (%)	402/234/9 (62/37/1)
APRI grade (1/2/3), n (%)	225/284/136(35/44/21)
CDS grade (1/2/3), n (%)	225/388/32 (35/60/5)
CTP classification (A/B-C), n (%)	605/40 (94/6)
FIB-4 grade (1/2/3), n (%)	135/282/228 (21/44/35)
GUCI grade (1/2/3), n (%)	220/288/137 (34/45/21)
ICG (1/2/3), n(%)	380/206/59 (59/31/9)
King’s score(1/2/3), n (%)	112/207/326 (17/32/51)
Lok index grade (1/2/3), n (%)	447/154/44 (69/24/7)
MELD score (<8/8–12/>12), n (%)	419/190/36 (65/29/6)
Serum Na (1/2), n (%)	616/49 (95/5)
PALBI grade (1/2/3), n (%)	372/213/60 (58/33/9)
Extent of hepatic resection	
Sub-segmentectomy, n (%)	26 (4)
Segmentectomy, n (%)	133 (21)
Bi-segmentectomy, n (%)	232 (36)
Lobectomy, n (%)	254 (39)

ALBI, Albumin-bilirubin; ALT, Alanine aminotransferase; AST, Aspartate aminotransferase; APRI, Aspartate transaminase-to-Platelet ratio; CDS, Cirrhosis discriminant index;

CTP, Child-Turcotte-Pugh score; FIB-4, Fibrosis-4 score; ICG, Indocyanine green;

HBV, hepatitis B virus; HCV, hepatitis C virus; MELD, Model for End-stage liver disease;

GUCI, Göteborg University Cirrhosis Index; PALBI, platelet-albumin-bilirubin;

SD, standard deviation; IQR, interquartile range.

### Tumor recurrence

**Figure 1:**
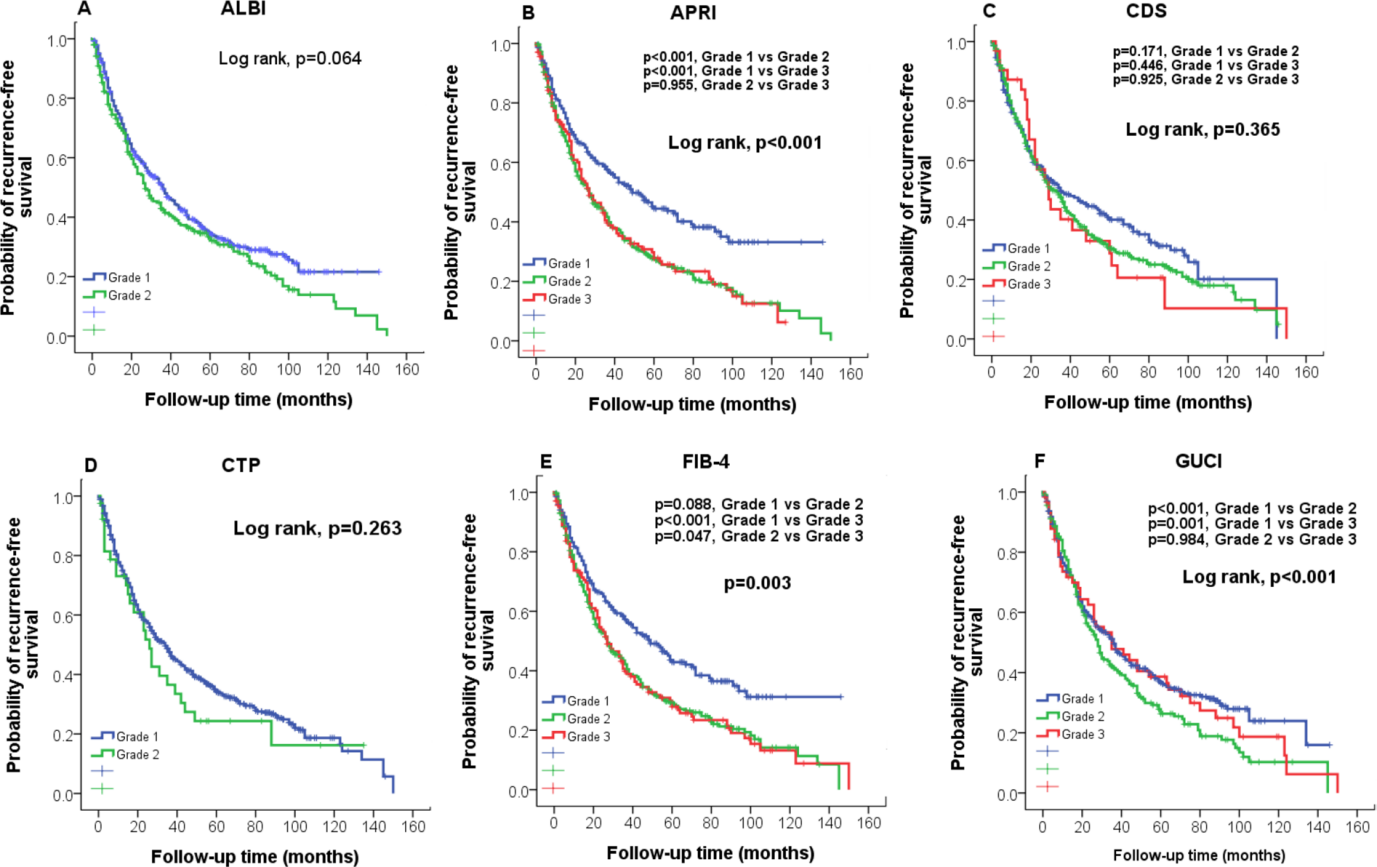
Comparison of recurrence-free survival distribution according to (**A**) ALBI, (**B**) APRI, (**C**)CDS, (**D**) CTP, and (**E**) FIB-4, (**F**) GUCI grading. Significant survival differences are found in APRI, FIB4 and GUCI (p < 0.05).

**Figure 2:**
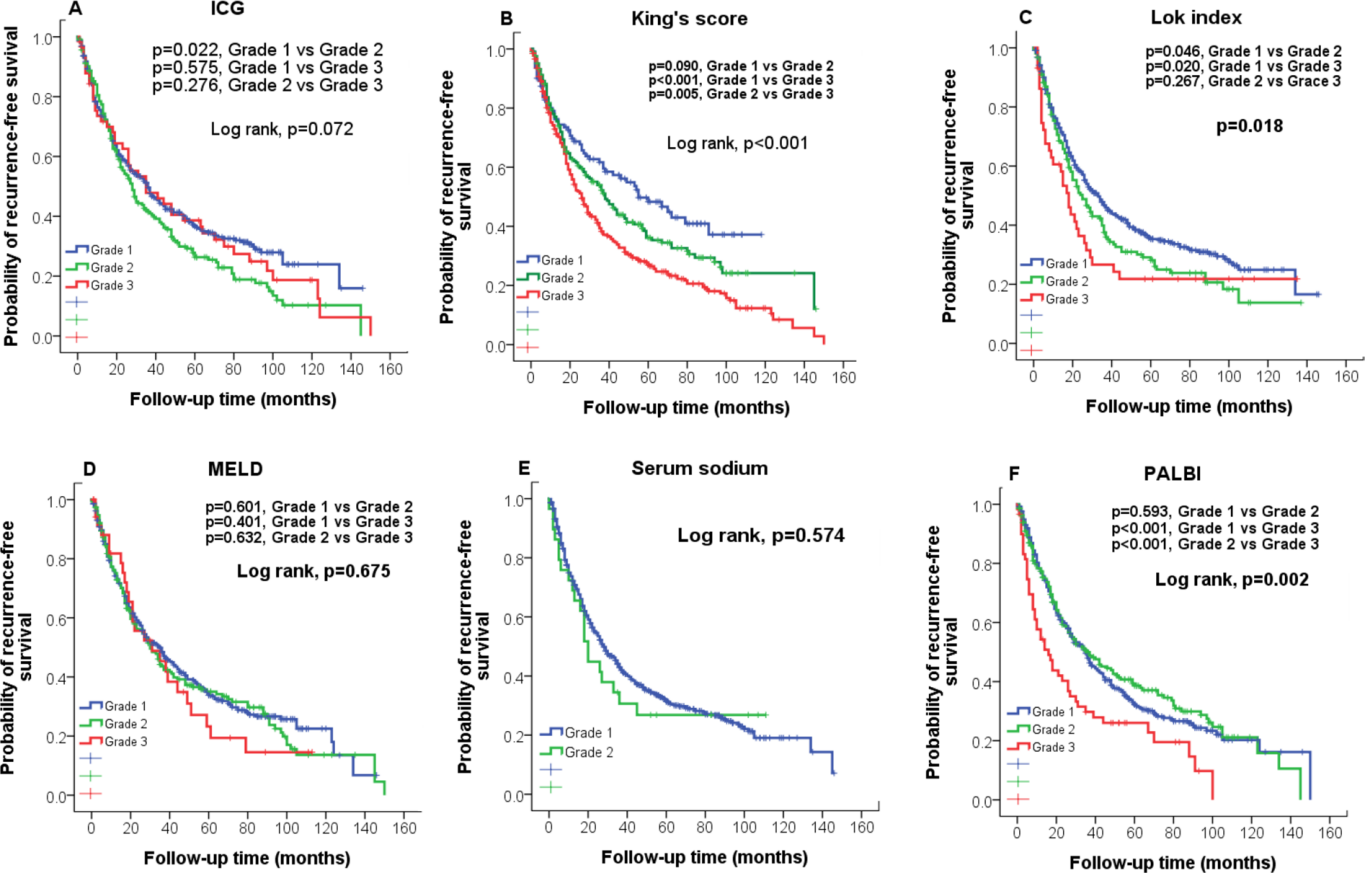
Comparison of recurrence-free survival distributions according to (**A**) ICG, (**B**) King’s score, (**C**) Lok index, (**D**) MELD, and (**E**) Serum sodium, (**F**) PALBI grading. Significant survival differences are found in King’s score, Lok index and PALBI (p < 0.05).

The median recurrence-free survival was 23 months, and 413 (64%) patients had tumor recurrence during the follow-up. The estimated 1-, 3-, and 5-year recurrence-free survival rates were 73%, 43% and 33%, respectively. The predictive role of 12 noninvasive liver functional reserve models on recurrence-free survival was evaluated according to their grading (Figs 1 and 2). A significant difference in recurrence-free survival were found only in APRI, FIB-4, GUCI, King’s score, Lok index and PALBI (all p < 0.05). Pairwise comparison showed that there was no significant difference between APRI grade 2 vs 3 (p = 0.995), GUCI grade 2 vs 3 (p = 0.984), Lok index grade 2 vs 3 (p = 0.267) and PALBI grade 1 and grade 2 (p = 0.593). Comparison of prognostic performance in terms of tumor recurrence prediction among 12 models reveals that the King’s score had the highest homogeneity and lowest AICc value (Table 3).

**Table 3:** Predictive accuracy of tumor recurrence and overall survival in 12 noninvasive liver functional reserve models.

	Homogeneity (Wald χ^2^)	Corrected Akaike information criteria (AIC)
Tumor recurrence		
ALBI	6.166	4968.555
APRI	17.027	4957.694
CDS	2.378	4972.344
CTP	0.535	4974.187
FIB-4	13.176	4961.545
GUCI	15.276	4959.445
ICG-15	6.025	4968.696
King’s score	21.248	4953.474
Lok index	6.186	4968.536
MELD	1.264	4973.457
Serum Na	0.298	4974.423
PALBI	4.831	4968.891
Overall survival		
ALBI	14.822	3999.685
APRI	3.724	4010.784
CDS	0.244	4014.263
CTP	1.496	4013.011
FIB-4	5.754	4008.753
GUCI	3.378	4011.130
ICG-15	3.944	4010.563
King’s score	7.663	4006.845
Lok index	5.736	4008.815
MELD	0.908	4013.600
Serum Na	3.334	4009.173
PALBI	8.491	4006.017

In univariate analysis, positive HBsAg, alcoholism, high AFP, tumor number > 3 nodules, tumor size > 3 cm, presence of vascular invasion and King’s score grade 3 were associated with increased risk of recurrence (all p < 0.05; Table 4). In the Cox model, 4 independent predictors of tumor recurrence were identified: alcoholism (HR: 1.443; 95% CI: 1.062–1.960, p = 0.019), high AFP level (HR: 1.499; 95% CI: 1.185–1.897, p = 0.001), tumor size > 3 cm (HR: 1.562, 95% CI: 1.219–2.001, p < 0.001) and King’s score grade 3 (HR: 1.770, 95% CI: 1.318–2.378, p < 0.001).

**Table 4:** Univariate and multivariate analysis of factors associated with tumor recurrence and overall survival.

**Tumor recurrence**	Number	Univariate analysis			Multivariate analysis		
		HR	CI	*p*	HR	CI	*p*
Age (<65/≥65 years)	399/246	1.030	0.845–1.255	0.769			
Sex (male/female)	518/127	0.912	0.712–1.68	0.465			
HBsAg (negative/positive)	235/410	1.251	1.020–1.535	0.032			
Anti-HCV (negative/positive)	490/155	1.028	0.822–1.286	0.811			
Alcoholism (no/yes)	552/93	1.379	1.062–1.789	0.016	1.443	1.062–1.960	0.019
DM (no/yes)	514/131	0.915	0.714–1.172	0.481			
Ascites (absent/present)	600/45	1.185	0.796–1.764	0.402			
Alpha-fetoprotein (<20/≥20 ng/mL)	301/344	1.399	1.152–1.699	0.001	1.499	1.185–1.897	0.001
Tumor nodules ( ≦ 3/ > 3nodules)	633/12	1.869	0.927–3.767	0.080			
Tumor size ( ≦ 3 cm/ > 3 cm)	230/415	1.374	1.121–1.684	0.002	1.543	1.203–1.978	0.001
Performance status (0/1–4)	507/138	1.287	1.016–1.629	0.036			
Vascular invasion (no/yes))	577/68	1.780	1.309–2.421	<0.001			
King’s score							
Grade 1	112	1			1		
Grade 2	207	1.205	0.876–1.658	0.252	1.327	0.958–1.838	0.088
Grade 3	326	1.741	1.297–2.337	<0.001	1.770	1.318–2.378	<0.001
Overall survival							
Age (<65/≥65 years)	399/246	1.283	1.036–1.589	0.023			
Sex (male/female)	518/127	0.941	0.716–1.236	0.661			
HBsAg (negative/positive)	235/410	0.854	0.687–1.061	0.153			
Anti-HCV (negative/positive)	490/155	1.130	0.889–1.436	0.319			
Alcoholism (no/yes)	552/93	1,346	1.010–1.794	0.043			
DM (no/yes)	514/131	1.429	1.108–1.841	0.006	1.489	1.152–1.925	0.002
Ascites (absent/present)	600/45	1.621	1.098–2.391	0.015			
Alpha-fetoprotein (<20/≥20 ng/mL)	301/344	1.695	1.365–2.106	<0.001	1.513	1.211–1.891	<0.001
Tumor nodules ( ≦ 3/ > 3 nodules)	633/12	2.816	1.496–5.298	0.001	2.599	1.372–4.924	0.003
Tumor size ( ≦ 3 cm/ > 3 cm)	230/415	2.035	1.602–2.584	<0.001	1.747	1.365–2.236	<0.001
Performance status (0/1–4)	507/138	1.589	1.228–2.058	<0.001	1.311	1.006–1.710	0.045
Vascular invasion (no/yes)	577/68	2.964	2.217–3.963	<0.001	2.334	1.723–3.162	<0.001
ALBI grade							
Grade 1	402	1			1		
Grade 2–3	243	1.526	1.231–1.891	<0.001	1.439	1.158–1.790	0.001

### Treatment after tumor recurrence

During the follow-up period, 377 (58%) patients had intrahepatic recurrence and 36 (9%) had extrahepatic recurrence. Treatment of recurrent HCC included re-resection (n = 35), local ablative therapy (n = 131), transarterial chemoembolization (n = 187), sorafenib (n = 6), chemotherapy (n = 16), radiotherapy (n = 10) and best supportive care (n = 27).

### Overall survival

The median overall survival was 55 months and 343 (53%) of patient died during follow-up. The cause of death was tumor recurrence in 213 (62.2%) patients. Another 73 (20.2%) patients died of liver failure or complications of portal hypertension with (66 patients) or without (7 patients) tumor recurrence, and the remaining 57 (16.6%) died of non-liver related causes.

**Figure 3:**
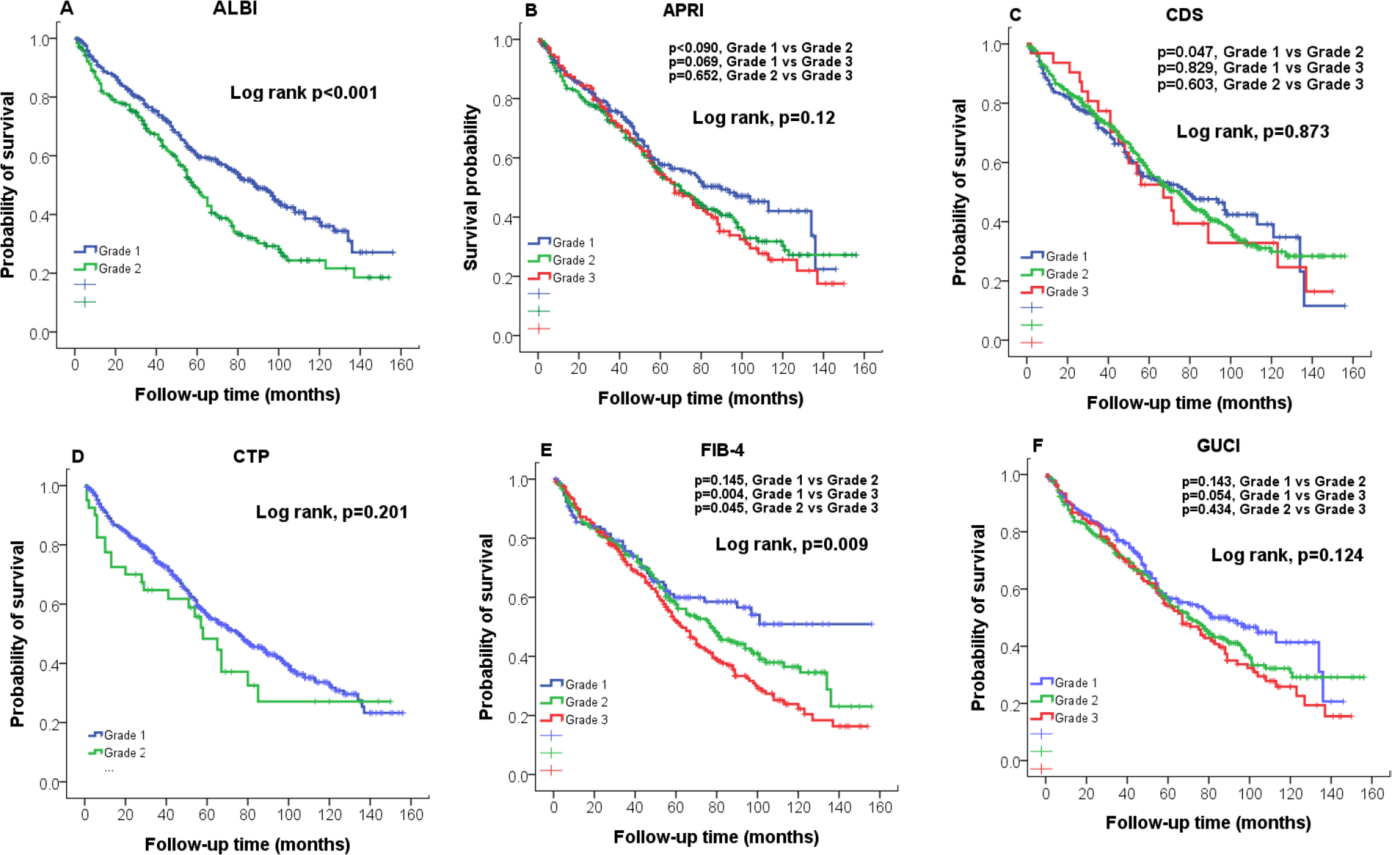
Comparison of overall survival distribution according to (**A**) ALBI, (**B**) APRI, (**C**)CDS, (**D**) CTP, and (**E**) FIB-4, (**F**) GUCI grading. Significant survival differences are found in ALBI and FIB-4 (p < 0.05).

**Figure 4:**
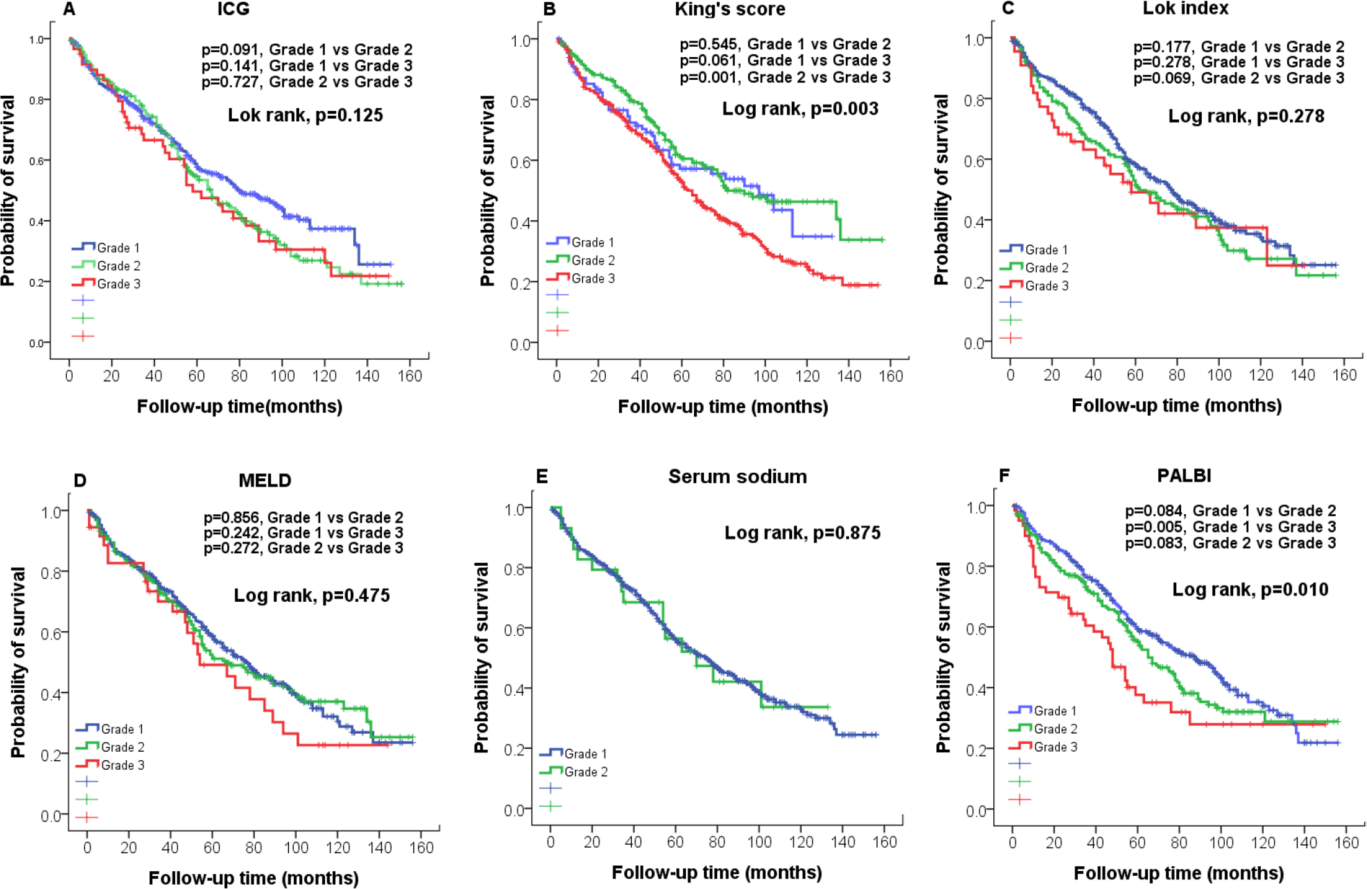
Comparison of overall survival distributions according to (**A**) ICG, (**B**) King’s score, (**C**) Lok index, (**D**) MELD, and (**E**) Serum sodium, (**F**) PALBI grading. Significant survival differences are found in King’s score and PALBI (p < 0.05).

The estimated 1-, 3-, and 5-year survival rates were 88%, 74% and 56%, respectively. The survival distribution according to the grading of 12 noninvasive liver reserve models are shown in Figs 3 and 4. Significant differences in overall survival were found only in ALBI, FIB-4, King’s core and PALBI (all p < 0.05). Pairwise comparison showed that there were no significant differences in FIB-4 grade 1 vs 3 (p = 0.145), King’s score grade 1 vs 3 (p = 0.545), PALBI grade 1 vs grade 3 (p = 0.084) and grade 2 vs grade 3 (p = 0.083). The prognostic role of these 12 models for survival analysis showed that the ALBI grade had the highest homogeneity and lowest AICc value (Table 3).

In univariate analysis of overall survival among surgical patients, older age (≥65 years), alcoholism, presence of diabetes mellitus, presence of ascites, high AFP level, larger tumor (>3 cm) and multi-nodularity (>3 nodules), poor performance status, presence of vascular invasion and ALBI grade 2–3 were associated with decreased survival (all p < 0.05, Table 4). In the Cox model, seven independent adverse prognostic predictors were found: diabetes mellitus (HR: 1.489, 95% CI: 1.152–1.925, p = 0.002), AFP ≥ 20 ng/ml (HR: 1.513; 95% CI: 1.211–1.891, p < 0.001), >3 tumor nodules (HR: 2.599, 95% CI: 1.372–4.924, p = 0.003), main tumor size > 3 cm (HR: 1.747, 95% CI: 1.365–2.236, p < 0.001), poor performance status (HR: 1.311, 95% CI: 1.006–1.710, p = 0.045), vascular invasion (HR: 2.334; 95% CI: 1.723–3.162, p < 0.001), and ALBI grade 2–3 (HR: 1.439, 95% CI: 1.158–1.790, p < 0.001).

### Correlation analysis

Except for serum sodium, the scores of other 10 noninvasive liver functional reserve models all significantly increased with higher ICG-15 value (Table 5).

**Table 5:** Correlation between ICG-15 and different liver functional reserve models.

ICG-15 retention rate (%)					
	<10	10–15	15–20	> 20	*p*
ALBI (Mean ± SD)	−3.81 ± 0.45	−3.69 ± 0.46	−3.53 ± 0.041	−3.31 ± 0.57	<0.001
APRI (Mean ± SD)	0.84 ± 1.23	1.26 ± 1.90	1.45 ± 1.47	1.66 ± 1.22	<0.001
CDS (Mean ± SD)	4.78 ± 1.46	5.02 ± 1.36	5.55 ± 1.65	5.98 ± 1.70	<0.001
FIB-4 (Mean ± SD)	2.62 ± 2.36	3.85 ± 3.95	4.67 ± 2.90	5.33 ± 2.80	<0.001
GUCI (Mean ± SD)	0.98 ± 1.50	1.44 ± 2.08	1.76 ± 1.84	2.05 ± 1.71	<0.001
Lok index (Mean ± SD)	0.39 ± 0.21	0.42 ± 0.19	0.50 ± 0.23	0.56 ± 0.24	<0.001
MELD (Mean ± SD)	7.83 ± 2.01	8.03 ± 1.73	8.49 ± 2.47	8.89 ± 2.89	<0.001
PALBI (Mean ± SD)	−2.60 ± 0.35	−2.55 ± 0.32	−2.52 ± 0.26	−2.34 ± 0.40	<0.001
King’s score (Mean ± SD)	22.41 ± 31.00	36.74 ± 52.79	47.91 ± 52.07	49.98 ± 39.67	<0.001
CTP (Mean ± SD)	5.25 ± 0.53	5.29 ± 0.60	5.46 ± 0.68	5.80 ± 1.35	<0.001
Serum sodium (mmole/L; Mean ± SD)	139.71 ± 2.64	139.65 ± 2.61	139.40 ± 3.18	138.90 ± 2.74	0.181

ALBI, Albumin-bilirubin; APRI, Aspartate transaminase-to-Platelet ratio; CDS, Cirrhosis discriminant index; CTP, Child-Turcotte-Pugh score; FIB-4, Fibrosis-4 score; MELD, Model for End-stage liver disease; GUCI, Göteborg University Cirrhosis Index; PALBI, platelet-albumin-bilirubin; SD, standard deviation.

## Discussion

Liver functional reserve is a crucial prognostic predictor for HCC. In this study, we utilize a prospective HCC cohort to evaluate the prognostic role of these noninvasive models on tumor recurrence and overall survival in HCC patients undergoing surgical resection. We show that among these noninvasive models, the King’s score is a more feasible marker to predict tumor recurrence and ALBI is the most accurate model in the discrimination of survival for HCC patients.

In the prediction of tumor recurrence, our results disclose that the King’s score, APRI and FIB-4 are the three most accurate models associated with recurrence according to AICc analysis. Among these models, the King’s score has the greatest homogeneity of recurrence pattern among HCC patients, indicating it is a more useful tool for recurrence prediction. In multivariate Cox model, King’s score grade 3 had 77% increased risk of recurrence as compared with those with grade 1. In addition to King’s score, tumor size, high AFP and alcoholism are also independent predictors associated with tumor recurrence. These findings suggest that liver functional reserve and tumor status are closely linked with a more aggressive tumor behavior.

In survival analysis, consistent with previous report^12,13,34^, we found that the ALBI and PALBI grade are the best models for discriminating patient survival. We further show that ALBI grade has the highest homogeneity for survival prediction, suggesting that ALBI is a more robust tool for outcome prediction. In multivariate Cox model, ALBI grade 2–3 was associated with 43% increased risk of mortality compared with ALBI grade 1.

Our analyses indicate that tumor size and number are closely related to survival of HCC patients. In addition, in accordance with previous studies^35,36^, performance status and vascular invasion are crucial prognostic predictors. Moreover, consistent with published series^37–40^, high serum AFP level and diabetes mellitus may strongly impact the outcome of HCC patients. Taken together, the severity of liver reserve, tumor burden and performance status are the hallmarks for survival prediction.

The CTP classification has been used to evaluate the severity of liver function and prognosis of HCC. However, in our study, CTP was not significantly linked with tumor recurrence and overall prognosis. This is probably because the majority (94%) of the patients were CTP class A and hence its prognostic ability is impaired. The MELD score and serum sodium level are used to evaluate the prognosis of end-stage cirrhotic patients in the process of organ allocation in liver transplantation^8,14^. However, these two models could not accurately discriminate tumor recurrence and survival because the patients in this study are mostly mildly cirrhotic or non-cirrhotic. Serum ICG-15 has been a useful adjunct to quantify hepatic reserve in HCC, but its performance is not superior to other markers in this study. Other noninvasive models (CDS, GUCI and Lok index) have not been used to evaluate the prognosis of HCC patients. Importantly, of all models, the ALBI grade, based simply on serum albumin and bilirubin level, is more objective and a readily available marker that can be used for survival discrimination in surgical HCC patients.

Among the 12 liver functional reserve models, APRI, FIB-4 and King’s score are principally designated as liver fibrosis models. Previous studies showed that these models could be used to predict the prognosis of HCC^41–43^. Notably, these models are associated with liver fibrosis which might be associated with an increased risk of tumor recurrence via carcinogenesis pathway. Interestingly, of these models, the King’s score is the best in predicting tumor recurrence in HCC patients undergoing surgical resection. Other noninvasive liver reserve models (CDS, GUCI and Lok index) have not been used to assess liver reserve and prognosis in HCC. Alternatively, the PALBI grade, an updated version of ALBI classification, is a new promising prognostic tool for HCC and more studies are required to validate its clinical role.

The correlation between ICG-15 and other noninvasive liver functional reserve models was investigated in this study. Our results show that, except for serum sodium, there is a strong correlation between ICG-15 and other 10 models, indicating that most models are clinically relevant in evaluating the degree of liver injury.

Liver functional reserve plays an important role in determining the extent of surgical resection for HCC. The major surgical resection was performed in patients who had good liver functional reserve. However, for those with relatively poor liver reserve, limited surgical resection was done, and these patients might have a higher risk of recurrence after surgery. As a result, liver function may have indirect impact on tumor recurrence via the choice of extent of surgical resection.

There are some study limitations. This is a single-center study in an HBV endemic area, thus external validation is needed from other study groups. In addition, the results are based on HCC patients undergoing surgical resection, therefore the prognostic accuracy of ALBI and King’s score in patients receiving different treatment modalities needs further study to establish. Lastly, since our hospital is a tertiary medical center, referral bias cannot be completely avoided.

In conclusion, the currently used liver functional reserve models have differential predictive ability for HCC patients undergoing surgical resection.The King’s score may serve as a feasible model in predicting tumor recurrence, whereas ALBI grade is the best prognostic tool among the 12 noninvasive liver reserve models. Appropriate models should be considered to integrate into cancer staging in future clinical practice.
